# Elementary and Practical Treatise on Internal Pathology

**Published:** 1845-04

**Authors:** 


*Elementary and Practical Treatise on Internal Pathology.* By A. Grisolle, &c. &c.

